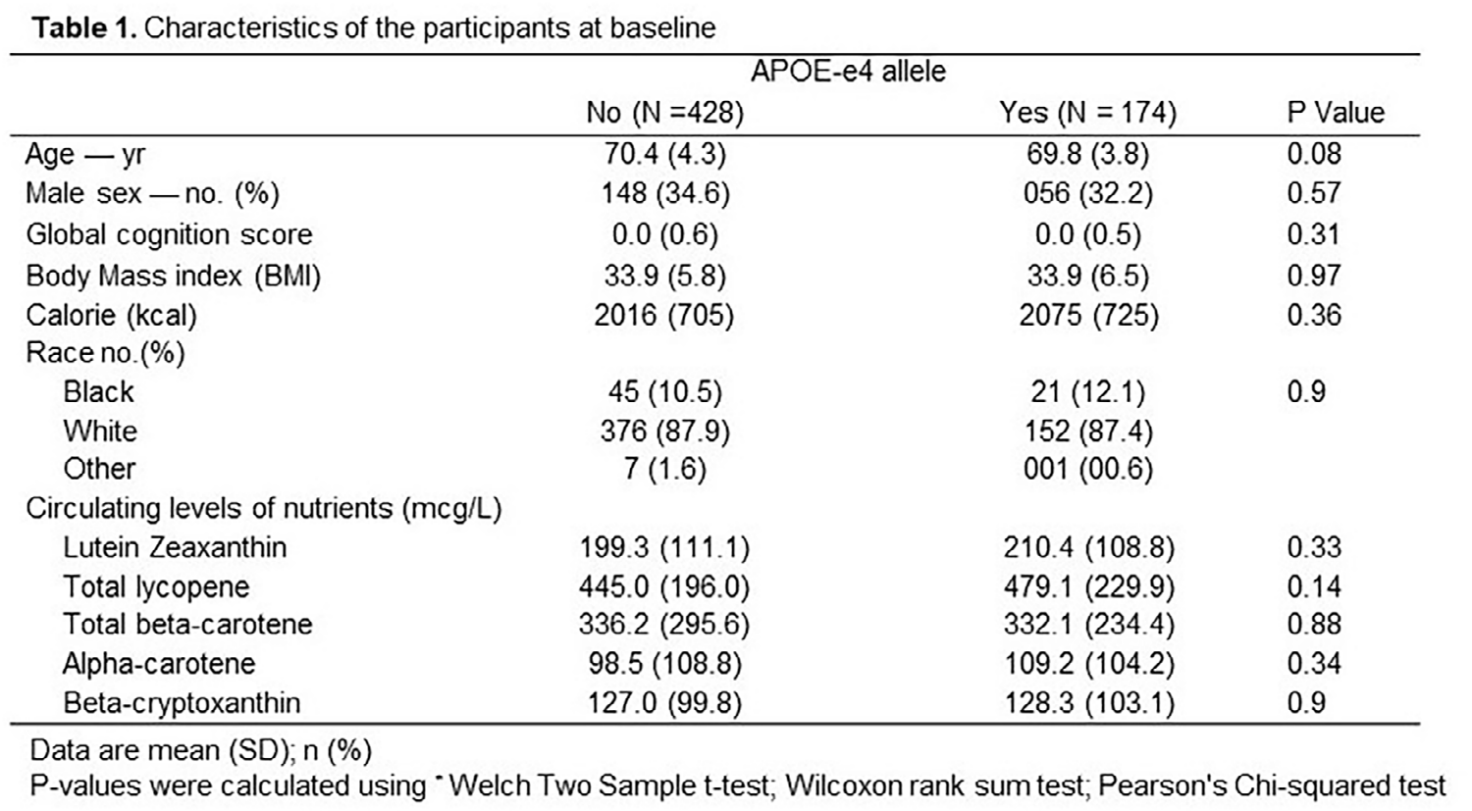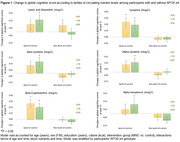# Higher plasma antioxidant nutrient levels associated with slower cognitive decline among individuals with APOE‐e4: an investigation of the MIND trial

**DOI:** 10.1002/alz.090784

**Published:** 2025-01-09

**Authors:** Xiaoran Liu, Kumar B Rajan, Todd Beck, Christy C Tangney, Klodian Dhana, Walter C. Willett, Neelum T. Aggarwal, Frank Sacks, Lisa L. Barnes

**Affiliations:** ^1^ Rush University Medical Center, Chicago, IL USA; ^2^ Rush Institute for Healthy Aging, Chicago, IL USA; ^3^ Rush University, Chicago, IL USA; ^4^ Harvard T. H. Chan School of Public Health, Boston, MA USA; ^5^ Rush Alzheimer’s Disease Center, Chicago, IL USA

## Abstract

**Background:**

Observational studies have shown a protective association between antioxidant nutrients and lower risk of Alzheimer’s disease. Results from dietary intervention trials remain inconclusive. The effect of APOE‐e4, the strongest risk factor for AD, on responsiveness to dietary interventions remains largely uninvestigated. We examined whether the association of plasma nutrient levels and changes in global cognition over three years differed by the APOE‐e4 allele among MIND trial participants.

**Method:**

The study included 601 participants from the MIND trial with APOE‐e4 genotype. Plasma nutrients were analyzed using HPLC at Harvard School of Public Health. Cognitive function was evaluated with a 12‐test battery from which a composite of global cognition was derived. Mixed‐effect regression model was used to examine the association of plasma nutrient levels, the APOE‐e4 allele and cognitive decline.

**Result:**

The mean age was 70.4 years for the 428 APOE‐e4 *non*‐carriers and 69.8 years for the 174 carriers (p = 0.08) (**Table 1**). Among APOE‐e4 carriers, individuals in the highest tertile of lutein and zeaxanthin, alpha‐carotene, and beta‐carotene had a significantly slower cognitive decline by 0.16 standardized unit (SU) [SE = 0.07, p = 0.03], 0.16 SU [SE = 0.07, p = 0.02], and 0.18 SU [SE = 0.07, p = 0.01], respectively, when compared with the lowest tertile (**Figure 1**). Among APOE‐e4 carriers, individuals in both the moderate and the highest tertiles of lycopene (0.24 ± 0.07, p = 0.001, 0.19 ± 0.07, p = 0.01) and beta‐cryptoxanthin (0.26 ± 0.07, p = 0.000, 0.20 ± 0.07, p = 0.01) had slower cognitive decline than those in the lowest tertile

Among APOE‐e4 *non‐*carriers, there was no association, except individuals in the moderate tertile of lutein and zeaxanthin which showed slower declines (0.08 ± 0.04, p = 0.04) compared to the lowest tertile.

When comparing APOE‐e4 carriers and *non*‐carriers at high plasma levels (75^th^ percentile) of alpha‐tocopherol, lutein and zeaxanthin, beta‐carotene, lycopene, and beta‐cryptoxanthin, APOE‐e4 carriers had slower global cognitive decline ranging from 4.7% to 21% (p<0.05 for all).

**Conclusion:**

Higher plasma antioxidant nutrients were associated with significantly slower cognitive decline among APOE‐e4 carriers in the MIND trial participants, suggesting the potential effects of antioxidant nutrients in mitigating the genetic risk posed by APOE‐e4.